# Alphavirus Replicon Particles Expressing TRP-2 Provide Potent Therapeutic Effect on Melanoma through Activation of Humoral and Cellular Immunity

**DOI:** 10.1371/journal.pone.0012670

**Published:** 2010-09-10

**Authors:** Francesca Avogadri, Taha Merghoub, Maureen F. Maughan, Daniel Hirschhorn-Cymerman, John Morris, Erika Ritter, Robert Olmsted, Alan N. Houghton, Jedd D. Wolchok

**Affiliations:** 1 Swim Across America Laboratory, Immunology Program, Sloan-Kettering Institute, New York, New York, United States of America; 2 AlphaVax, Inc., Research Triangle Park, North Carolina, United States of America; 3 Ludwig Institute for Cancer Research, New York Branch, New York, New York, United States of America; New York University, United States of America

## Abstract

**Background:**

Malignant melanoma is the deadliest form of skin cancer and is refractory to conventional chemotherapy and radiotherapy. Therefore alternative approaches to treat this disease, such as immunotherapy, are needed. Melanoma vaccine design has mainly focused on targeting CD8^+^ T cells. Activation of effector CD8^+^ T cells has been achieved in patients, but provided limited clinical benefit, due to immune-escape mechanisms established by advanced tumors. We have previously shown that alphavirus-based virus-like replicon particles (VRP) simultaneously activate strong cellular and humoral immunity against the weakly immunogenic melanoma differentiation antigen (MDA) tyrosinase. Here we further investigate the antitumor effect and the immune mechanisms of VRP encoding different MDAs.

**Methodology/Principal Findings:**

VRP encoding different MDAs were screened for their ability to prevent the growth of the B16 mouse transplantable melanoma. The immunologic mechanisms of efficacy were investigated for the most effective vaccine identified, focusing on CD8^+^ T cells and humoral responses. To this end, ex vivo immune assays and transgenic mice lacking specific immune effector functions were used. The studies identified a potent therapeutic VRP vaccine, encoding tyrosinase related protein 2 (TRP-2), which provided a durable anti-tumor effect. The efficacy of VRP-TRP2 relies on a novel immune mechanism of action requiring the activation of both IgG and CD8^+^ T cell effector responses, and depends on signaling through activating Fcγ receptors.

**Conclusions/Significance:**

This study identifies a VRP-based vaccine able to elicit humoral immunity against TRP-2, which plays a role in melanoma immunotherapy and synergizes with tumor-specific CD8^+^ T cell responses. These findings will aid in the rational design of future immunotherapy clinical trials.

## Introduction

Melanoma differentiation antigens (MDAs) include tyrosinase, pMEL17/gp100, gp75/tyrosinase related protein (TRP)-1, MART-1/melan-A and dopachrome tautomerase/TRP-2 and represent ideal target antigens for melanoma immunotherapy, due to preferential expression in melanocytes and melanoma cells [1]. Although eliciting immune responses against MDAs has been challenging because self tolerance hampers complete activation of adaptive immunity [2], recent progress with melanoma vaccines has provided proof of principle that T cell immunity to MDAs can be actively induced in advanced melanoma patients. However, established tumors develop an array of immune-escape mechanisms that inhibit effector T cells and/or prevent full T cell activation [3], [4], limiting the clinical benefit. The notion the tumor immune-escape mechanisms are heterogeneous and multifaceted has challenged the idea of a central and unique role for CD8^+^ T cells in tumor immunotherapy. An emerging concept is that targeting multiple arms of immunity may be important to counteract tumor immune-escape at different levels. Indeed, it has recently been shown that targeting regulatory T cells or effector CD4^+^ T cells is therapeutic [5], [6], [7] suggesting that combined activation/deactivation of different T cell populations might be beneficial.

Activation of humoral responses may represent further improvement of current T cell-based melanoma immunotherapy strategies. Monoclonal antibodies targeting tumor surface antigens such as HER2/neu, CD20, EGF receptor and CD52, for example, are effective for the treatment of other malignancies, through immune cell-dependent and independent mechanisms [8]. The development of melanoma-specific humoral immunity to numerous surface and intracellular melanoma antigens, including MDAs, naturally occurs in melanoma patients [9], [10], [11]. The recent observation that antigen-specific B cell immune responses correlate with durable objective clinical responses and stable disease in metastatic melanoma patients undergoing immunotherapy [12], suggests that IgGs may play a role in immune surveillance. A monoclonal antibody recognizing mouse gp75/TRP-1 has been generated and has shown activity in mice when infused either as single agent or in combination with vaccines eliciting CD8^+^ T cell responses [13], [14]. However, inducing anti-tumor humoral immunity against MDAs through active vaccination has been a more elusive goal and the anti-tumor potential of humoral immunity has not been thoroughly appreciated in melanoma [15], [16].

Recombinant viral vectors have been investigated as a means of vaccination because they can carry full-length antigen-encoding genes, have the capacity to produce these antigens in large quantities, and may contain helper epitopes. Alphaviruses are positive-stranded RNA viruses. An attenuated variant of one member of this family, the Venezuelan equine encephalitis virus (VEE), has been developed as a propagation-defective virus-like replicon particle (VRP). VEE-based VRP have been shown to induce high titers of antibodies and robust antigen-specific T cell responses against encoded antigens in mice [17], [18], [19], [20], [21], [22], [23] and more recently in healthy human subjects [24]. At the same time, neutralizing anti-vector immunity does not appear to preclude benefit from repetitive booster vaccinations in mice [25], [26], [27], [28], [29] as opposed to other viral vectors. VRP generate double-stranded RNA that stimulates innate immune responses through RNA binding receptors [22], [30], [31], potent inducers of innate antiviral responses, including type I IFNs [32].

An alphavirus-based DNA vaccine encoding gp75/TRP-1 induces immunity to both mouse and human gp75/TRP-1 [21]. Similarly, we have shown that VRP containing either syngeneic (mouse) or human tyrosinase are immunogenic as opposed to plasmid DNA vaccination, which requires the use of xenogeneic (human) tyrosinase to overcome immunologic ignorance and/or tolerance [33]. Interestingly, VRP can elicit both antibodies and CD8^+^ T cell responses against tyrosinase.

Given the very attractive ability of VRPs to induce humoral and T cell responses against tyrosinase, we sought to further investigate the potentials of VRP encoding different MDAs as vaccines for melanoma. We found that VRP-encoded TRP-2 (VRP-TRP2) is the most effective target antigen among those tested. The VRP-TRP2 vaccine has a surprisingly potent therapeutic anti-tumor effect as a single agent on B16 tumors. We also found that the efficacy of VRP-TRP2 relies on its ability to elicit TRP-2 specific antibodies. Here we report for the first time that TRP-2 specific antibodies, induced by active immunization, synergistically co-operate to tumor protection together with CD8^+^ T cells.

## Results

### Screening the best MDA encoded by VRPs in a prophylactic setting

**Figure 1:**
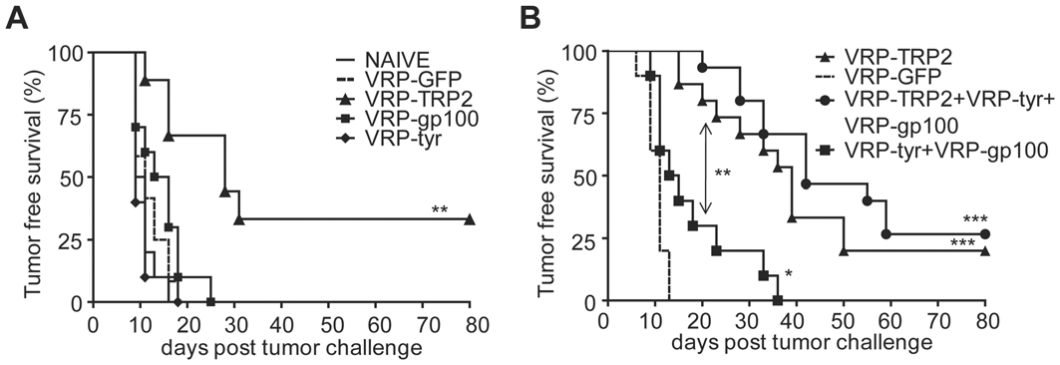
VRP-TRP2 has a potent anti-tumor effect in a prophylactic setting. Kaplan-Meier tumor-free survival curves are shown for 10/15 mice per group. Mice received i.d. injections of VRP expressing TRP-2, gp100, tyrosinase (**A**) or combination of those antigens (**B**) prior to challenge with B16 melanoma (prophylactic setting). As a negative control, groups of mice were injected with VRP-GFP or left untreated (NAÏVE). *** P<0.0001, ** P<0.001, * P<0.01.

To investigate the efficacy of VRPs encoding MDAs as an immunotherapeutic approach for melanoma, we compared VRP encoding the mouse MDAs tyrosinase (tyr), gp100 or TRP-2. VRP were administered in a prophylactic setting, prior to intradermal (i.d.) challenge with the aggressive and poorly immunogenic B16 melanoma line. Shown in Figure 1A, mice vaccinated with VRP-gp100 and VRP-tyr developed tumor within 18 days, similar to untreated mice or control mice injected with VRP expressing green fluorescent protein (GFP, VRP-GFP). However, the VRP-TRP2 vaccine provided a significant delay in tumor occurrence and long-term tumor protection (Figure 1A). Vaccination with VRP-TRP2 was more effective than combination of VRP-gp100 and VRP-tyr, and the efficacy of the combination of all three VRP expressing the different melanoma antigens was not significantly better than VRP-TRP2 alone (Figure 1B). Depigmentation was observed in only in 5–10% of immunized animals. These results suggested that TRP2 is the most relevant antigen to target with VRP in a prophylactic setting; therefore, we focused on this vaccine and further characterized its preclinical potential by testing it in more stringent therapeutic experimental conditions.

### VRP-TRP2 in a therapeutic setting

**Figure 2:**
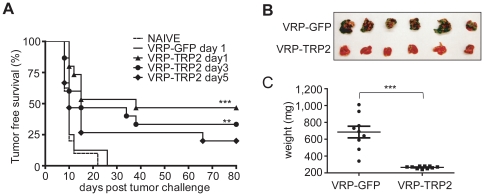
VRP-TRP2 has a potent anti-tumor effect in a therapeutic setting. (**A**) Kaplan-Meier tumor-free survival curves are shown for 10/15 mice per group. Mice received i.d. injections of VRP expressing the indicated MDAs either 1or 3 days after challenge with B16 melanoma (therapeutic setting). As a negative control, groups of mice were injected with VRP-GFP or left untreated (NAÏVE). (**B**) Representative photograph and (**C**) weight of lungs resected 24 days after tumor inoculation from mice treated with VRP-GFP or VRP-TRP2 (lung therapeutic model). Each dot represents an individual mouse. *** P<0.0001, ** P<0.001.

The anti-tumor effect of VRP-TRP2 treatment was evaluated in a more clinically relevant therapeutic setting. To this end, mice were injected with B16 to establish skin tumors prior to receiving vaccination with VRP-TRP2. VRP-TRP2 induced time-dependent tumor protection when vaccination was started as late as 5 days after tumor inoculation (Figure 2A). Similar therapeutic potency was observed in a different challenge model, where B16 is injected intravenously (i.v.) and develops as tumor nodules in the lung. Mice treated with VRP-TRP2 developed fewer lung tumors when compared to control mice (Figure 2B and 2C). These data indicated that TRP-2-specific immune responses developed rapidly in the VRP-TRP2 vaccinated animals and were effective in regressing tumors in different anatomic locations. Although prophylaxis against B16 implantation can be achieved by many types of vaccines, eradication of B16 tumors after tumor implantation can only be accomplished by the combination of vaccination with other treatment modalities [34]. In the cutaneous therapeutic setting, tumors develop very rapidly and reach a size of 1 cm^2^ at day 25–35 in close to 100% of untreated mice. The surprising therapeutic effect of the VRP-TRP2 vaccine as monotherapy in un-manipulated B16 tumor with these stringent conditions, prompted further investigation of the mechanism(s) underlying tumor immunity.

### VRP-TRP2 induces TRP-2 specific CD8+ T cell responses and accumulation of CD8+ T cells in the tumor

**Figure 3:**
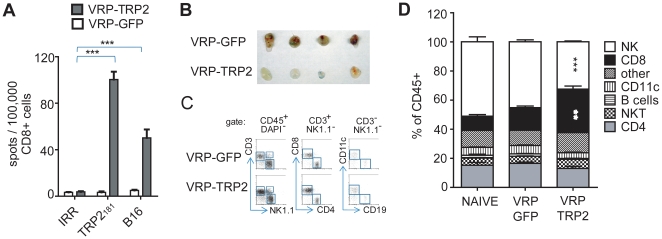
VRP-TRP2 elicits a tumor-specific CD8^+^ T cell responses. (**A**) ELISPOT analysis of IFNγ secreting CD8^+^ T cells purified from spleens of mice immunized with control VRP-GFP or VRP-TRP2 and re-stimulated with TRP-2_181_ peptide, B16 cells or an irrelevant peptide (IRR). Mean value and standard error represents 3/5 spleens analyzed individually per group. (**B–D**) Flow cytometric analysis of immune cell lineages infiltrating B16-matrigel plugs. (**B**) Representative photograph of B16-matrigel plugs of mice vaccinated with control VRP-GFP or VRP-TRP2 and (**C**) representative dot plot. (**D**) Immune infiltrate analysis expressed as a percentage of total CD45^+^ leukocytes. Mean percentage and standard error represent 4/5 tumors analyzed individually per group.

It has been shown that the anti-tumor effect of TRP-2 specific DNA vaccines is CD8^+^ T cell dependent [35], [36]. We therefore first considered the possibility that the anti-tumor activity of VRP-TRP2 may depend on the activation of TRP-2 reactive CD8^+^ T cells. To investigate this, we performed *ex vivo* and *in vivo* analyses of CD8^+^ T cell responses. Measurement of IFNγ production by ELISPOT assays and intracellular flow cytometry staining showed that immunization with VRP-TRP2 induced CD8^+^ T cells that were responsive to the TRP-2_181–188_ peptide (Figure 3A and Figure S1A). VRP-TRP2 induced CD8^+^ T cells also recognized B16 cells as a target *in vitro* (Figure 3A). VRP-TRP2 induced a stronger CD8^+^ T cell response against the TRP-2_181_ immunodominant epitope when compared to the reactivity elicited by VRP-gp100 and VRP-tyr against the gp100_25_ and tyr_360_ immunodominant epitopes, respectively (Figure S1B).

We measured the quality and quantity of the immune infiltrate in B16 tumors implanted after vaccination. The percentage of tumor infiltrating CD45^+^ cells significantly increased when mice were immunized with VRP-TRP2 as compared to VRP-GFP or naïve mice (Figure S2). The quality of the CD45^+^ immune tumor infiltrate also selectively changed with an increase in the percentage of CD3^+^CD8^+^ T cells recruited at the tumor site, indicating that B16 reactive CD8^+^ T cells were trafficking to tumor *in vivo* (Figure 3B-D). A significant decrease in tumor infiltrating NK cells was also observed in VRP-TRP2 vaccinated mice, whereas the percentage of T cells, B cells, NKT cells and CD11c^+^ dendritic cells remained unchanged (Figure 3D).

### CD8^+^ T cells are important but are not the only cell type responsible for tumor protection

**Figure 4:**
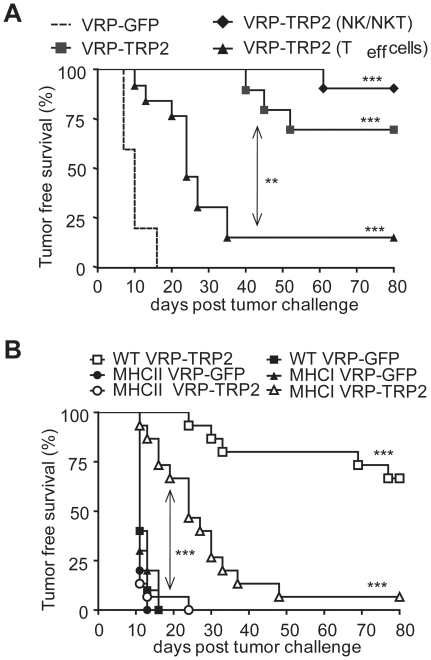
VRP-TRP2 anti-tumor effect is only partially CD8^+^ T cell dependent. (**A**) Kaplan-Meier tumor-free survival curves of 10 mice per group vaccinated with either control VRP-GFP or VRP-TRP2 and treated with depleting antibodies for NK/NKT or T cells after the last vaccination. (**B**) Kaplan-Meier tumor-free survival curves of 15 WT, MHC I and MHC II deficient mice per group vaccinated with either control VRP-GFP or VRP-TRP2. *** P<0.0001, ** P<0.001.

In order to determine which components of the immune system contribute to the anti-tumor effect induced by the vaccine, we depleted effector T cells (both CD4^+^ and CD8^+^), NK and NKT cells with depleting antibodies in vivo, after vaccination. Despite the consistent recruitment of CD8^+^ T cells to tumor (Figure 3D), depletion of effector T cells after vaccination only partially decreased tumor-free survival upon B16 challenge (Figure 4A). Depletion of NK and NKT cells did not affect the anti-tumor effect, ruling out a possible contribution of these cells in VRP-TRP2 mediated anti-tumor immunity (Figure 4A). To further investigate the role of T cells in tumor protection we immunized MHC I deficient mice, which lack CD8^+^ T cells. VRP-TRP2 immunized MHC I deficient mice showed a lower degree of protection than that observed in control wild-type (WT) mice, but had a significantly better outcome as compared to similar mice immunized with control VRP-GFP (Figure 4B). This indicated that the anti-tumor effect of VRP-TRP2 does not only rely on effector CD8^+^ T lymphocytes. To investigate the role of other immune components we tested the VRP-TRP2 vaccine in MHC II deficient mice, which lack CD4^+^ T cells and did not mount TRP2-specific CD8^+^ T cell responses (Figure S3). The anti-tumor effect of VRP-TRP2 was completely abrogated in MHC II deficient mice (Figure 4B). Interestingly, MHC I deficient mice showed a better degree of protection than MHC II deficient mice (Figure 4B), implying a CD4^+^ T cell-dependent CD8^+^ T cell-independent mechanism of tumor protection.

### TRP-2 expressing VRPs induce TRP2-specific IgGs

Since the simultaneous depletion of CD4^+^ and CD8^+^ effector T cells after vaccination did not completely abrogate tumor protection (Figure 4A), we hypothesized that CD4^+^ T cells may contribute to the antitumor effect of VRP-TRP2 through the activation of B cell responses and therefore investigated a role for humoral immunity.

**Figure 5:**
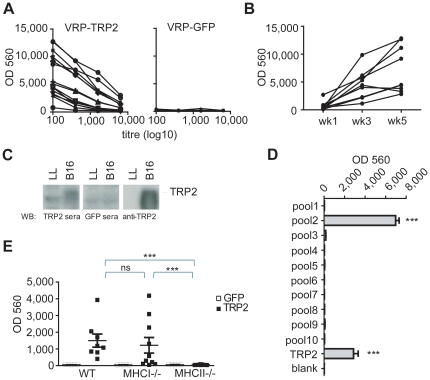
VRP-TRP2 elicits a TRP2-specific antibody response. (**A,B**) Measurement of TRP-2-specific serum IgG by ELISA. (**A**) Serial dilutions of sera from mice immunized with VRP-TRP2 (left panel) or VRP-GFP (right panel). Each line represents an individual mouse. (**B**) 1∶100 dilution of sera from mice immunized with VRP-TRP2 one week after the first (week 1), the second (week 3) or the third vaccination (week 5). Each line represents an individual mouse. (**C**) Western blot analysis of sera from VRP-TRP2 or VRP-GFP immunized mice and a commercial TRP-2 specific antibody (anti-TRP2) against B16 or Lewis Lung (LL) cell lysates. (**D**) Measurement of TRP-2-peptide specific serum IgG by ELISA. 1∶100 dilution of sera from mice immunized with VRP-TRP2 were tested against the indicated TRP-2 peptide pools. (**E**) 1∶100 dilution of sera drawn from WT, MHC I or MHC II deficient mice immunized with VRP-TRP2 or VRP-GFP were analyzed. Each dot represents sera from individual mice. *** P<0.0001.

Mice immunized with VRP-TRP2 developed TRP-2 specific serum IgG that recognized recombinant TRP-2 protein by ELISA (Figure 5A, left panel). This was not observed in mice immunized with the control VRP-GFP vaccine (Figure 5A, right panel). The titer of TRP-2 specific IgG increased over time following repetitive vaccinations (Figure 5B). Sera from VRP-TRP2 immunized mice also recognized endogenous murine TRP-2 expressed by B16 cells, as assessed by Western blot analysis on tumor protein extracts (Figure 5C). By using a library of overlapping peptides (Figure S4) we found that the serum from VRP-TRP2 immunized mice was preferentially reactive to a region spanning amino acids 51–101 of mouse TRP-2 (Figure 5D). The induction of TRP-2-specific IgG was entirely MHC II dependent but did not require MHC I (Figure 5E), suggesting that TRP-2 specific IgG may indeed account for the residual anti-tumor activity of VRP-TRP2 observed in MHC I deficient mice (Figure 4B).

### The humoral response induced by VRP-TRP2 is important for tumor protection and requires Fc receptors (FcR)

**Figure 6:**
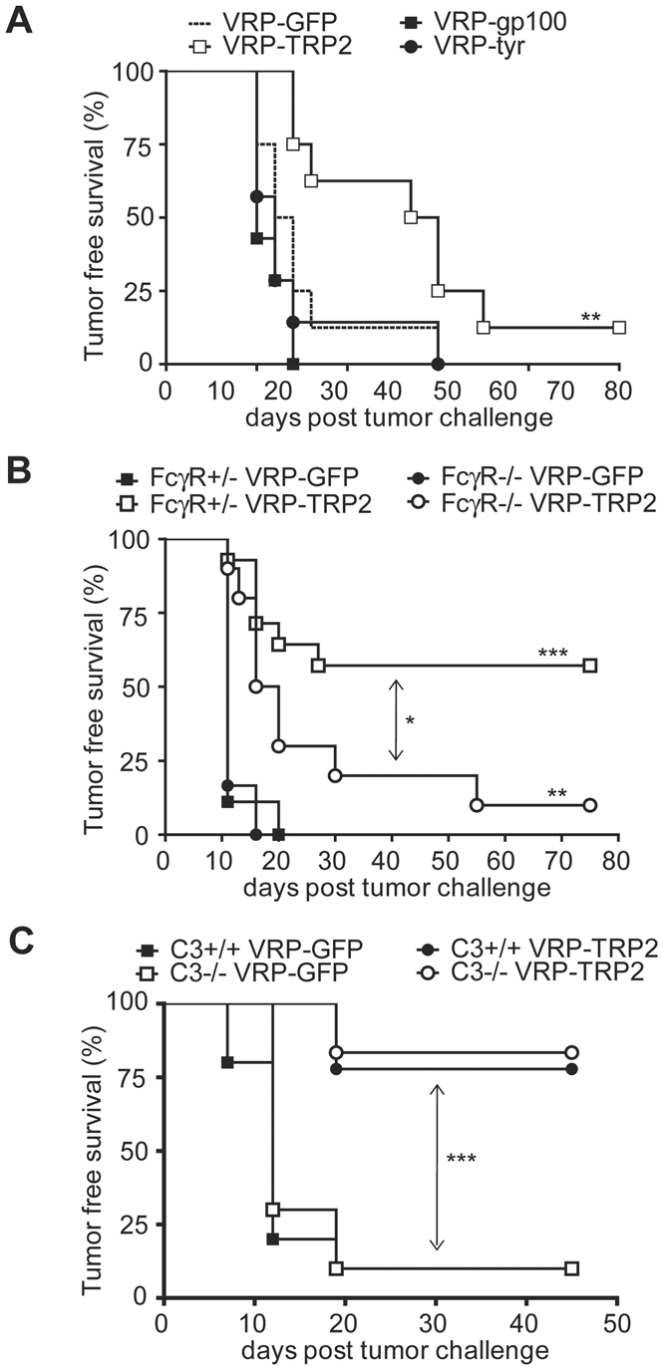
VRP-TRP2 humoral anti-tumor effect is mediated by Fc Receptors. Kaplan-Meier tumor-free survival curves of (**A**) 7–9 NAÏVE recipient mice per group, transferred with sera from donor mice vaccinated with the indicated VRP; (**B**) Eight to 14 FcγR −/− or +/− littermate control mice vaccinated with VRP-TRP2 or VRP-GFP; (**C**) Twelve C3 −/− or +/+ control mice vaccinated with VRP-TRP2 or VRP-GFP. *** P<0.0001, ** P<0.001, * P<0.01.

To evaluate whether the TRP-2 antibody response plays a role in tumor protection, sera from mice immunized with VRP were transferred to recipient mice that were challenged on the same day with B16. VRP-TRP2 immune sera delayed tumor growth when compared with sera from mice immunized with VRP expressing other antigens (Figure 6A). To further investigate the IgG-dependent mechanism underlying the anti-tumor effect, mice deficient in the common γ chain (FcRγ^−/−^) were vaccinated with VRP-TRP2. FcRγ^−/−^ mice were less protected compared with FcRγ^+/−^ littermate controls (Figure 6B). These results indicated that the antibody-dependent anti-tumor effect involves signaling through activating Fc receptors. FcRγ^−/−^ and FcRγ^+/−^ controls induced similar levels of TRP-2 specific IgG (Figure S5); therefore, the reduced tumor protection is likely due to the lack of IgG effector function and not to inherent inability of FcRγ^−/−^ mice to activate humoral responses. By contrast, C3^−/−^ animals showed a similar level of tumor protection as WT controls (Figure 6C), ruling out a major role for complement in the anti-tumor effect.

## Discussion

Heterogeneity of immune escape mechanisms leads to the suggestion that targeting multiple arms of immunity could be a key to the development of more potent immunotherapies. Most pre-clinical and clinical studies of melanoma vaccines have focused on eliciting tumor-specific CD8^+^ T cells, which can be very effective in preventing tumor occurrence but may not be sufficient to control tumor growth when the tumor is already established. Here we have evaluated the efficacy of non replicative alphavirus-based as vaccines for melanoma and we have shown that (1) the choice of the target antigen determines the therapeutic outcome and (2) the mobilization of humoral immunity is an important step in optimizing the efficacy of TRP-2 specific melanoma vaccines.

We have first found that a VRP vaccine targeting TRP-2 is surprisingly effective in controlling tumor growth as opposed to vaccines based on the same viral delivery vector targeting the other melanosomal antigens, gp100 and tyrosinase. Therefore, although TRP-2, gp100 and tyrosinase are all MDAs expressed in the melanosome, their relative immunogenicity and therapeutic potential is different, at least when delivered via VRPs. In addition to its intrinsic immunogenicity, the relevance of TRP-2 as a target antigen may also relate to the TRP-2 protein's localization and function. TRP-2 is a transmembrane melanosomal glycoprotein required for melanin biosynthesis and its expression is restricted mainly to the melanocyte, melanoblasts and retinal pigmented epithelium [1], [37], [38], [39]. Although TRP-2 expression in normal melanocytes is targeted to the melanosomes, the polypeptide matures in the endoplasmic reticulum and a small proportion of TRP-2 can be found in the plasma membrane [40], [41], therefore providing a target for antibodies.

TRP-2 has DOPAchrome tautomerase enzymatic activity and catalyzes the conversion of DOPAchrome to 5,6,dihydroxyndole-2-carboxylic acid, a precursor of brown melanins. It has been proposed that TRP-2 may maintain cell viability by controlling the intracellular concentration of 5,6-dihydroxyndole, a toxic intermediate of melanin biosynthesis [42]. Mice with mutations in the TRP-2 gene have a lighter color coat (Slaty) as compared to wild type C57BL/6 mice, due to decreased catalytic function [43]. In addition, TRP-2 is expressed in migratory melanoblasts early after they emerge from the neural crest, whereas other MDAs, such as tyrosinase and TRP-1 are expressed later in development [37]. Early expression of TRP-2 in melanogenesis may also contribute to its relevance as a tumor immunotherapy target given the heterogeneity in differentiation state of individual melanomas [10].

It has been previously reported that TRP-2 specific CD8^+^ T cell responses can be induced in humans and in mice [35], [44]. Here we demonstrate for the first time that TRP-2 specific humoral immunity induced by VRP vaccination is another important component of active immunization to achieve long term tumor protection. Indeed, although either tumor specific CD8^+^ T cells or IgG alone are sufficient to provide a significant short term tumor protection, both are required for long term protective immunity. This suggests that activating both arms of immunity is an important requirement to subvert immune-escape mechanisms against established and rapidly growing tumors such as the poorly immunogenic B16 melanoma. The ability of the alphavirus-based VRP vaccine to achieve this dual immune effect may rely on the high amounts of antigen produced and/or on its adjuvant effect on innate immune cells. *In vitro* experiments have shown that the RNA component of the VRP delivery system contributes to its immunogenicity by triggering inflammatory signals through RNA binding receptors such as RIG-I and MDA-5 [21], [22], [30], [31]. The activation of cytoplasmic RNA sensing pathways in specific antigen presenting cell subsets in vivo may therefore be an important requirement for an adjuvant to overcome tolerance to self antigens and prime multifaceted immune responses.

We have shown that the anti-tumor activity of VRP-TRP2 is in part mediated by signaling through activating Fc receptors and not through C3a-receptor. This suggests that antibody-dependent cell mediated cytotoxicity (ADCC) rather than complement mediated lysis, is important for the in vivo anti-cancer effect. It is emerging that Fc receptors play an important role in modulating in vivo anti-tumor toxicity of therapeutic monoclonal antibodies [45]. TRP-2 directed Fc-dependent ADCC may require direct killing of IgG coated targets by Mac-1 expressing neutrophils or macrophages [46], [47]. Alternatively, FcR-mediated uptake of dying tumors may result in cross presentation and induction of a broader-spectrum immune protection. The two possibilities are not mutually exclusive and will require further investigation.

Immunization against MDAs can cause skin hypopigmentation, as consequence of autoimmune reactions to normal melanocytes [9]. Tumor protection induced by prophylactic immunization with mouse TRP-2 can correlate to different degrees of hypopigmentation depending on the vaccination regimen [36], [48]. TRP-2 may also have antigenic potential in humans, as a significant anti-TRP2 antibody titer was observed in patients with vitiligo [49]. To our surprise, we found that VRP-TRP2 induced very little hypopigmentation in mice, confined to the site of tumor injection. This feature is attractive, in that the potency of this vaccine does not seem to correlate with severe autoimmune side effects in mice.

Our preclinical work has identified a new potent therapeutic vaccine that provides long-term protection against melanoma through concomitant activation of antibodies and CD8^+^ T cells targeting the self antigen TRP-2. Our studies demonstrate for the first time that targeting TRP-2 with both humoral and cellular immunity is a valuable approach to enhance the efficacy of melanoma vaccine on established tumors. These findings will be instrumental in designing future immunotherapeutic approaches for melanoma and support further investigation on the efficacy of VRP-TRP2 in melanoma patients.

## Materials and Methods

### Mice and cell lines

All mouse procedures were performed in accordance with institutional protocol guidelines at Memorial Sloan-Kettering Cancer Center (MSKCC). C57BL/6J (8–10-wk-old females) and C3 deficient mice (strain B6.129S4-C3tm1crr/J) were obtained from the Jackson Laboratory. MHC class I deficient (strain B2MN12), MHC class II deficient (strain ABBN12) and WT control mice (strain B6NTac) were purchased from Taconic Farms. Mice deficient in the FcR common γ chain, provided by J. Ravetch (Rockefeller University, New York, New York) were backcrossed onto WT C57BL/6 and bred at MSKCC. Mice were maintained according to NIH Animal Care guidelines, under a protocol 96-04-017 approved by the MSKCC Institutional Animal Care Committee. The B16-F10 mouse melanoma line was originally obtained from I. Fidler (M.D. Anderson Cancer Center, Houston, TX).

### VRP preparation

Mouse tyrosinase, gp100 and TRP-2 cDNAs were cloned into the pERK replicon vector and VRP were generated as previously described [50].

### VRP immunization and tumor challenge

C57BL/6 mice were vaccinated three times at two week intervals with 10^6^ VRP diluted in PBS by subcutaneous (s.c.) injection into the plantar surface of each footpad. In prophylactic tumor protection experiments, mice were challenged with 7.5×10^4^ B16F10 cells intradermally (i.d.) two weeks after the last vaccination with VRP. For T cell depletion experiments, CD4+ cells, CD8+ cells and NK/NKT cells were depleted by intraperitoneal injection of 200 µg of GK1.5, 2.43 and PK136 antibodies (Monoclonal Antibody Core Facilty, MSKCC) respectively, at day −11, −4, +4, +11 relative to tumor inoculation. In therapeutic tumor protection experiments, mice were first challenged with 7.5×10^4^ B16F10 cells either i.d. (cutaneous therapeutic model) or i.v. (lung therapeutic model), and then vaccinated weekly for three times. Animals were monitored every 2 to 3 days for 80 days. For the Kaplan-Meier tumor free survival curves, mice were considered tumor free until tumors were visible or palpable. P values were calculated with log-rank (Mantel-Cox) test.

### T cell assays

Spleens were harvested 5 to 7 days after the last VRP immunization and CD8^+^ T cells were positively selected for standard ELISPOT assays as previously described [14]. Flow cytometry based intracellular staining assays were performed on CD8^+^ T cells purified from the spleen and then incubated overnight with monensin prior to staining with the fixation and permeabilization kit (eBioscience) using anti-CD8-PE–Texas red, anti-CD3-FITC, anti-IFNγ-APC (BD) and LIVE/DEAD Fixable Aqua Dead Cell Stain kit (ViD). TRP2_181–189_, gp100_25–33_, tyr_360–368_ and the overlapping peptide library were synthesized by Genemed Synthesis at >80% purity.

### Analysis of the tumor infiltrate

Seven days after B16-matrigel s.c. injection (1×10^5^ B16-F10 cells in 0.2 ml of Matrigel Matrix Growth Factor Reduced; BD), the matrigel plug was resected, incubated for 1 hour at 37°C with 1 mg/ml Collagenase D (Sigma) and dissociated to obtain a single-cell suspension. Cells were stained with anti-CD45.2 PercpCy5.5, anti-CD3-FITC, anti-NK1.1-APC, anti-CD8-PE–Texas red, anti-CD4- Alexa Fluor 700 (BD) and DAPI.

### ELISA and Western blot

Seven days after the last VRP immunization, mice were bled from the lateral tail vein or via cardiac puncture. Sera were analyzed by ELISA using plates coated with a purified recombinant TRP2 protein in PBS and goat anti mouse IgG-AP (Southern Biotech), as a detection antibody. The AttoPhos AP substrate was purchased from Promega (#S1013). Plates were read with Perceptive Biosystems, Cytoflour Series 4000 at Excitation 450/50 and Emission 580/50 with gain of 25. Western blotting was performed using sera pooled from immunized mice and diluted 1∶100 in PBS+0.1% Tween20 and incubated overnight at 4°C.

### Passive transfer of immune sera

Sera were collected from mice 7 days after the last VRP immunization. 50 ul of sera were injected i.v. in recipient mice at day 0, 3 and 6. Recipient mice were challenged with 7.5×10^4^ B16 cells i.d. at day 0 and tumor occurrence was monitored as described above.

## Supporting Information

Figure S1 CD8+ T cell antigen specific IFNgamma production. (A) Flow cytometric analysis of IFNgamma+CD8+ T lymphocytes purified from spleens of mice immunized with control VRP-GFP or VRP-TRP2, and re-stimulated with TRP-2181 peptide or an irrelevant peptide (IRR). (B) ELISPOT analysis of IFNgamma secreting CD8+ T cells purified from spleens of mice immunized with control VRP-GFP, VRP-TRP2, VRP-gp100 or VRP-tyr and re-stimulated with TRP-2181, gp10025, tyr360 or an irrelevant peptide (IRR). Data shown represents the average of 3 individual mice.
(5.17 MB TIF)

Figure S2 Analysis of CD45+ tumor immune infiltrate. Flow cytometric analysis of the B16-matrigel immune infiltrate. (A) Representative dot plots and gating strategy. (B) The percentage of CD45+ cells among total live (DAPI-) cells is reported. Each dot represents an individual tumor analyzed, mean and standard errors are reported. ** P<0.001.
(5.15 MB TIF)

Figure S3 Analysis of TRP2-specific CD8+ T cell in MHC II KO mice. ELISPOT analysis of IFNgamma secreting CD8+ T cells purified from spleens of mice immunized with control VRP-GFP or VRP-TRP2 and re-stimulated with TRP-2181 or an irrelevant peptide (IRR). Mean value and standard error represents 3 spleens analyzed individually per group. ** P<0.001.
(4.63 MB TIF)

Figure S4 Overlapping TRP2 peptide library. A library of 102 overlapping peptides spanning the whole sequence of the TRP-2 protein was divided in 10 pools, each containing the indicated peptides.
(6.21 MB TIF)

Figure S5 Analysis of TRP2-specific IgG responses in FcgammaR KO mice. Measurement of TRP-2 specific serum IgG by ELISA. 1∶100 dilution of sera drawn from FcgammaR−/− or FcgammaR+/− mice immunized with VRP-TRP2 or VRP-GFP were analyzed. Each dot represents sera from individual mice. ** P<0.01.
(4.44 MB TIF)
